# Heterogeneous intratumor irradiation: a new partner for immunotherapy

**DOI:** 10.1080/2162402X.2024.2434280

**Published:** 2024-11-26

**Authors:** Paul Bergeron, Fabien Milliat, Eric Deutsch, Michele Mondini

**Affiliations:** a INSERM U1030, Gustave Roussy, Université Paris-Saclay, Villejuif, France; b Institut de Radioprotection et de Sûreté Nucléaire (IRSN), PSE-SANTE/SERAMED/LRMed, Fontenay-aux-Roses, France

**Keywords:** Cancer, immunotherapy, low-dose radiotherapy, radiotherapy, SFRT, spatially fractionated radiotherapy

## Abstract

We recently demonstrated that a heterogeneous tumor irradiation strategy, combining high-dose and low-dose radiotherapy (RT) within the same tumor volume, can synergize with immunotherapy in mice. Our findings indicate that heterogeneous RT doses may promote the spatial diversification of the antitumor immune response. Spatial fractionation of the RT dose has the potential to enhance the therapeutic index of RT/IO combinations, particularly in scenarios where irradiating the entire tumor volume is unfeasible or excessively harmful to the patient.

## Main text

Traditionally, radiotherapy (RT) has aimed to homogeneously deliver the maximum tolerated radiation dose to the entire tumor volume. However, there are clinical situations where adhering to this principle may pose excessive risks to the patient due to RT-induced toxicities to the organs at risk (OARs).^1^ To mitigate RT toxicity, recent technological advancements have focused on optimizing various irradiation (IR) parameters, including the implementation of ultra-high RT dose rates (FLASH RT) and spatially heterogeneous RT dose distribution (spatially fractionated RT, SFRT). SFRT has recently reemerged as an alternative to conventionally fractionated RT (CFRT) in the field of RT/immunotherapy (IO) combinations. The aim of SFRT is to optimize the RT dose distribution within the tumor by delivering heterogeneous doses, specifically administering high-dose RT (HDRT) to smaller tumor volumes and low-dose RT (LDRT) to the remaining tumor tissues, thereby enhancing the protection of surrounding healthy tissue from radiation.^2^ Studies have shown that various methods of SFRT (e.g., GRID, LATTICE, minibeam) can achieve interesting results in both clinical and preclinical settings, while exhibiting limited toxicity.^2^ It is hypothesized that the immunological/biological efficacy of SFRT may depend on the alternation of high and low doses, although the exact mechanisms remain poorly understood. However, the reemergence of SFRT underscores the need to elucidate its biological implications, as well as the modifications it induces in the immune microenvironment of the tumor volumes treated with different doses.^2^

In our recent publication in *Nature Communications*, we developed a heterogeneous tumor irradiation strategy of mice tumors, combining HDRT and LDRT within the same tumor volume using the millimetric precision allowed by a small animal radiation research platform (SARRP).^3^ We demonstrated that this unconventional SFRT regimen combining HDRT (16 Gy) on one half of the tumor volume and LDRT (2 Gy) on the other half could synergize with PD-1 blockade and promote complete responses in murine colorectal tumors (MC38 and CT26).

**Figure 1.**
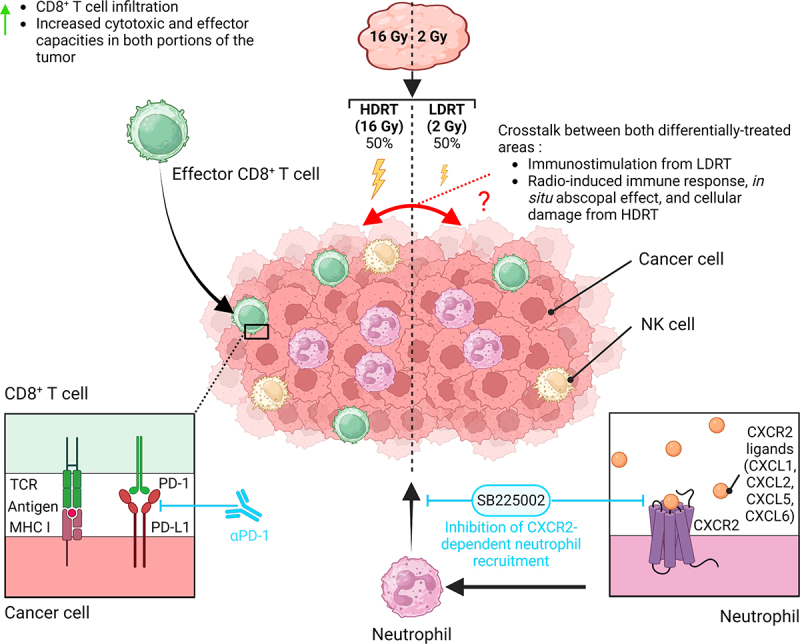
Non-homogeneous tumor irradiation reshapes the tumor immune microenvironment. Combining high (16 Gy) and low (2 Gy) radiation doses within the same tumor induced a spatially heterogeneous increased infiltration of CD8^+^ T cells, NK cells and neutrophils when compared to homogeneous RT, suggesting a potential crosstalk between the differentially treated areas. This non-homogeneous dose distribution favored the effector capacities of the CD8^+^ T cells, which could be further enhanced by PD-1 blockade. On the other hand, countering the intratumor infiltration of immunosuppressive CXCR2^+^ neutrophils with the addition of a CXCR2 antagonist (SB225002) led to the increased efficacy of the heterogenous IR plus anti-PD-1 combination. Created in BioRender. Bergeron, P. (2024). https://BioRender.com/u38e483.

In-depth flow cytometry and single-cell RNA sequencing characterization of the MC38 tumor immune microenvironment (TIME) suggested that the non-homogeneous regimen induced stronger intratumor infiltration (especially in the 16 Gy-treated volume) of effector immune populations such as CD8^+^ T and natural killer cells (NKs) compared to totally irradiated (TI) tumors (with 2 or 16 Gy). Additionally, analyses showed that CD8^+^ T cells from heterogeneously irradiated tumors exhibited stronger effector functions, which were further enhanced after anti-PD1 treatment (Figure 1). In addition, the analysis of differentially expressed genes suggested that CD8^+^ T cells from both HDRT and LDRT portions of these tumors were phenotypically less different from each other than CD8^+^ T cells coming from distinct TI tumors irradiated with 16 Gy and 2 Gy. Overall, our data suggest a pivotal role for activated CD8^+^ T cells and a bilateral crosstalk between the two portions of the heterogeneously irradiated tumors, which could contribute to the efficacy of the treatment when combined with PD1 blockade. These findings are in line with hypotheses from previous literature suggesting that the cytotoxic and immunogenic capacities of HDRT could synergize with the immunostimulating properties of LDRT in SFRT regimens.^2,4,5^

Of particular interest, a recent study by Jagodinsky and colleagues, which also employed heterogeneous RT doses, reached similar conclusions.^6^ They showed that heterogeneous RT dose administration in tumors using high-dose-rate brachytherapy could synergize with dual ICB (anti-PD-L1 and anti-CTLA4), outperforming homogeneous RT dose administration. They observed that such RT modality induced spatial heterogeneity in immune features, as well as increased effector functions in the CD8^+^ T cells, which were required (together with CD4^+^ T cells) for the antitumor efficacy of the treatment. Their findings suggest that low doses of RT are critical, as combinations of high and intermediate RT doses impaired antitumor efficacy. This study also highlights the interest of heterogeneous doses of RT for preserving functional antitumor immunity, as high IR doses could drastically reduce dendritic cells infiltration into the tumor-draining lymph nodes. In this context, focusing high-dose radiation on the remote portion of the tumor while minimizing exposure to surrounding lymph nodes could be an important for improving clinical outcomes.

The outcome of anticancer therapy at least partly relies on the balance between immunostimulatory and immunosuppressive signals.^7^ Notably, in our study, we found that the non-homogeneous regimens led to strong infiltration of immunosuppressive CXCR2^+^ neutrophils in the MC38 tumors. The increased efficacy of the non-homogeneous IR regimen plus anti-PD1 combination after the addition of a CXCR2 antagonist suggests that neutrophils may represent an interesting target for future investigations (Figure 1), as also suggested by previous studies.^8^

Further studies are needed to optimize heterogeneous IR modalities to fully capitalize on their promising immunomodulatory effects and enhance their synergistic efficacy with IO agents, in view of a translation in the clinical practice. Given that fractionated CFRT regimens have been shown to better synergize with IO agents than single-dose CFRT, it seems interesting to explore the temporal fractionation of the dose in SFRT. This could help normalize tumor vasculature, re-oxygenate the stroma, and facilitate effector immune cell infiltration.^9^

Unraveling the immune mechanisms induced by different SFRT modalities might help in identifying new biomarkers, leading to more personalized treatments in the clinical setting. In this regard, the development of spatial transcriptomics could be crucial for deeply characterizing the tumor stroma after heterogenous irradiations. Additionally, non-homogeneous stereotactic body RT may be a viable modality in clinical settings, as demonstrated in a clinical study by Korpics and colleagues.^8^ Moreover, the intratumor RT dose pattern can already be rationally designed with consideration for tumor heterogeneity in the clinics, as illustrated by approaches such as SBRT-PATHY,^10^ where the high RT doses are delivered to the hypoxic tumor segments. Of interest, the recent advances in imaging and radiomics^11^ offer a rationale for the personalized spatial adjustment of the RT dose gradient in the clinical setting, potentially leveraging structural and immunological heterogeneity within the tumor to elicit a diversified immune response. This approach could be readily transferred to the clinics, offering an opportunity to enhance the therapeutic index of RT/IO combinations, particularly in contexts where irradiating the entire tumor volume would be unfeasible or excessively harmful to the patient.
